# South African medical rehabilitation services delivery: a mixed method exploring the views of stakeholders on availability, accessibility, quality, affordability, equity, effectiveness, and efficiency

**DOI:** 10.11604/pamj.2024.48.159.43199

**Published:** 2024-08-07

**Authors:** Senzelwe Mazibuko, Thayananthee Nadasan, Pragashnie Govender

**Affiliations:** 1 Department of Physiotherapy, School of Health Sciences, College of Health Sciences, Westville Campus, University of KwaZulu-Natal, Glenwood, Durban, 4041, South Africa

**Keywords:** Medical rehabilitation, health services, availability, accessibility, quality, affordability, equity, effectiveness, efficiency, South African

## Abstract

**Introduction:**

South Africa has endeavored to provide access to rehabilitation to more people with the greatest needs via its National Rehabilitation Policy. To achieve the aim of the Framework and Strategy for Disability and Rehabilitation Services in South Africa, scientific knowledge on rehabilitation services would be essential to inform implementation, yet little is known in the KwaZulu-Natal (KZN) province. This paper describes South African rehabilitation services delivery focused on availability, accessibility, quality, affordability, equity, effectiveness, and efficiency based on stakeholders´ perspectives in KZN.

**Methods:**

a mixed method study design was employed to investigate 99 stakeholders' opinions on rehabilitation services in three districts of South Africa´s KZN province (eThekwini, Amajuba and King Cetshwayo) using focus group discussions, interviews, and a survey. The stakeholders included practitioners (physiotherapists, occupational therapists, social workers, psychologists, nutritionists, dietitians, audiologists, speech therapists, and bio kineticist), district rehabilitation managers, provincial rehabilitation managers, and representatives from the provincial office of social development. Whilst quantitative data were analysed using descriptive statistics, thematic analysis was employed for the qualitative data.

**Results:**

of the 99 participants, 73 (74%) were female, and ages ranged from 23 to 56 years (median=29 years, IQR=12.5 years). The emerging themes were availability, accessibility, equity, quality, efficacy, and efficiency of rehabilitation. Rehabilitation services were mostly (78%, n = 42) available at the tertiary level in urban areas, and few (45%, n = 42) in rural areas. No private rehabilitation facility was found in any rural setting. Accessibility was generally agreed to be poor (with patients traveling more than 10 kilometers to access services) but with the agreement that transport routes from surrounding areas were safe and reliable for patients to reach their setting (median=2.5, IQR=1.0). This study´s participant's general sentiment on affordability and equity tended to be mixed but skewed towards agreeing that the services were indeed affordable, and provided without any social class biases. Also, the general sentiment of the participants on the quality, effectiveness, and efficiency of rehabilitation services in their settings tended to be positive.

**Conclusion:**

rehabilitation at the primary level results in accessible services as they become closer to needy communities. We recommend prioritization of human resources and budgeting for rehabilitation services at the primary level to improve access to people in rural areas.

## Introduction

The South African Government´s Framework and Strategy for Disability and Rehabilitation Services in South Africa (FSDRS-SA) recognises the importance of rehabilitation in the continuum of healthcare and its essence to the achievement of a quality life [1]. Access to quality healthcare that is affordable drives economic development; however, disease pandemics, non-communicable diseases, and growing demand for improved health systems create complex and expensive challenges [2-4]. In 2018, the United Nations (UN) published a flagship report on realisation of the sustainable development goal for persons with disabilities [5]. It was noted that access to rehabilitation services remains a significant challenge, with more than 50% of persons with disabilities having an unmet need for rehabilitation services [5]. As a signatory of the United Nations Convention on the Rights of Persons with Disabilities (UNCRPD) [6], South Africa is increasing rehabilitation accessibility to make services geographically closer to most vulnerable clients and persons with disability (PWDs) [1]. Consequently, the FSDRS-SA brings into effect Article 26 of the UNCRPD which seeks to organise, strengthen, and extend access to rehabilitation services [1]. Rehabilitation should be available to those who need it. A study by the Human Science Research Council in 2013 suggests that the public sector is not providing effective, efficient, or equitable rehabilitation services [7,8]. The lack of rehabilitation services, and access to rehabilitation, is not dissimilar to that in other low- and middle-income countries (LMICs). The World Health Organization (WHO) launched the Global Rehabilitation 2030 Initiative, which aims to promote rehabilitation as a critical health strategy of the 21^st^ century worldwide [7,9,10]. This will assist countries in strengthening through multi-prolonged approaches, including an assessment process that culminates in strategic planning, as well as a rehabilitation-specific monitoring and evaluation framework [10].

As a developing nation, rehabilitation access in South Africa is often linked to socio-economic status [11]. Access and availability to rehabilitation are achieved if one can afford it. Rehabilitation clients living in rural areas face problems with accessing and using rehabilitation services [12]. Equity into proper rehabilitation access in rural areas has been hampered by poor funding, lack of patient involvement, travel distances, transport, and high financial costs [12]. Levels of care offered for rehabilitation ought to be aligned with what the community and country can afford. No individual should be denied rehabilitation because of an inability to pay. These challenges to improve the healthcare system demand that the South African government allocate resources. Public health is often challenged by fiscal shortages, constrained innovation, stagnant technological advancement, and poor human resources for healthcare [2-4]. South Africa´s life expectancy has improved significantly post-democracy, resulting in many more people living longer. However, non-communicable diseases such as diabetes, stroke, and cardiovascular injuries (CVI) have increased the number of rehabilitation clients, mostly in rural areas [12]. Rehabilitation averts the adverse complications of disability, disease, and serious injury through physical training and assisting with the social reintegration of the client [11-14]. In the South African government´s quest to re-engineer primary healthcare (PHC), rehabilitation has been identified as an integral feature of a transformed public health system [1].

Rehabilitation should be timeous and a practical link between medical treatment and the end-user´s successful restoration into a capacity of full social, economic, and overall productive life [1,11-14]. Rehabilitation service provision should be given as soon as it is required, be decentralised with coherent referral systems, and link community health centers to specialized tertiary institutions [1,11-14]. The organisational culture within the Department of Health, along with a historically silo-approach to service planning and delivery, and an often-narrow approach to healthcare delivery focusing on preventative and curative care offered by a limited number of health professionals constantly fails to recognise or include disability and rehabilitation in essential health policy and planning initiatives [15]. Many South Africans rely on the public sector for care and hence it is crucial to understand the current state of rehabilitation at all levels (national, provincial, district, and community) to ensure appropriate actions to integrate rehabilitation within National Health Insurance (NHI). This study aimed to describe the South African medical rehabilitation services delivery focused on availability, accessibility, quality, affordability, equity, effectiveness, and efficiency based on stakeholders´ perspectives in KZN.

## Methods

**Study design:** a mixed method study design was employed to investigate stakeholders' opinions on rehabilitation services (availability, accessibility, equity, quality, efficiency, and efficacy) in South Africa's KZN province using a focus group discussion (FGD), interviews, and a survey. A mixed method study design was chosen to comprehensively capture and analyze diverse stakeholders' opinions on rehabilitation services in KZN province, ensuring a rich understanding of both quantitative and qualitative aspects.

**Target study population:** this study targeted all rehabilitation stakeholders in three districts of KZN province that is eThekwini Metropolis, King Cetshwayo District Municipality, and the Amajuba District Municipality. These stakeholders included rehabilitation practitioners (physiotherapists, occupational therapists, dieticians/nutritionists, speech therapists, social workers, psychologists, and audiologists); and public health rehabilitation district; Department of Social Development (DoSD) and the Department of Health (DoH) representatives from KZN Provincial Office.

**Sample and recruitment:** a total of 99 participants were recruited from the three districts for this study (Table 1). Only three districts were included due to the logistical constraints of the project. Purposive sampling was used to recruit 57 rehabilitation practitioners and policymakers and implementers such as physiotherapists, occupational therapists, social workers, audiologists, speech therapists, psychologists, social workers, district rehabilitation managers, and representatives from KZN´s provincial DoSD and DoH. Stratified sampling was used to recruit 42 practitioners from all three districts who completed a researcher-designed survey.

**Table 1:** distribution of the study sample (N = 99)

Functional Level		District		Institution	Designation	No of participants
Implementation/service provision		Amajuba		Amajuba District Hospital	Practitioners focus group	15
Implementation/service provision		Amajuba		Mother & Child Hospital	Practitioners focus group	9
Implementation/service provision		Amajuba		District Health Office	Rehabilitation manager interview	1
Control/monitoring and evaluation		eThekwini Municipality		King Edward Hospital	Practitioners focus group	7
Implementation/service provision		King Cetshwayo District		Private rehab centre	Practitioners focus group	9
Implementation/service provision		King Cetshwayo District		Ngwelezane Hospital	Practitioners focus group	10
Control/monitoring and evaluation		King Cetshwayo District		District Office	Rehabilitation manager’s focus group interview	2
Control/monitoring and evaluation		King Cetshwayo District		Social Development District Office	Social development manager interviews	2
Policy/intelligent development		KwaZulu-Natal Province		Department of Health Provincial Office	Provincial rehabilitation managers interview	2
Distribution of the cross-sectional survey respondents (n = 42)						
**King Cetshwayo**		**Ethekwini District**		**Amajuba District**		
**Practitioner**	**n**	**Practitioner**	**n**	**Practitioner**	**n**	
Physiotherapist	8	Physiotherapist	3	Physiotherapist	5	
Occupational therapist	4	Audiologist	2	Occupational therapist	2	
Audiologist	3	Occupational therapist	1	Audiologist	2	
Psychologist	3			Social worker	1	
Dietitian	3			Dietitian	1	
Social worker	2					
Bio kineticist	2					

**Data collection:** qualitative data were collected through FGD and in-depth interviews by the principal researcher in the three included districts. Two one-on-one interviews with a representative from DoSD were conducted. Moreover, a dyad interview with a provincial health representative was conducted. Additionally, three FGDs with medical rehabilitation practitioners were conducted. Duration for the FGDs, and interviews, lasted between 30 and 60 minutes. All FGDs and interviews were audio-recorded and conducted in English. The qualitative design facilitated the in-depth examination of the stakeholders´ perceived availability, accessibility, equity, quality, efficiency, and efficacy of medical rehabilitation services in the three districts. The basic demographic profile of participants was also collected using a simple biographical survey. To ensure trustworthiness, no pre-existing relationships were created between researchers and participants before the start of the study. Two pilot sessions comprised of one FGD with practitioners and one interview with a District manager interview in a neighbouring district municipality were conducted to ensure rigour of the study methods.

Forty-two practitioners from all three districts completed a quantitative survey. The survey was designed by the authors, two physiotherapists, and an occupational therapist. Over four months, the authors went through an iterative process of modification. In addition, the process also included a review by an independent expert person in the discipline of physiotherapy, who is also a researcher and knowledgeable in biostatistics. The survey had six sections, Section A to F. Section A included a demographic profile of the sample group. Section B relates to current rehabilitation practice in terms of human resources. Closed-ended and multiple-choice items were used to gain an understanding of rehabilitation disciplines present at each institution; available support staff; doctor to patient ratio; and a five-item Likert scale ascertaining experience, service quality, salary satisfaction, and recruitment consultation. Section C was concerned with current referral pathway practice, systems, and protocol. Section D dealt with facility or institutional information. The section covers the geographical setting where the facility is situated; health sector serviced; available infrastructure; the use of the International Classification of Function (ICF) classification system, and the mode of rehabilitation session assessment. Quality control processes were covered in Section E. Sub-sections included closed-ended, multiple choice items on areas of rehabilitation service; rehabilitation clients serviced; and mode of administration. Section E included a 5-point, 17-item Likert scale on rehabilitation service quality control processes. The Likert scale considers the quality of service; ease of bureaucracy; maintenance; grievance queries, and feedback. Section F dealt with rehabilitation service delivery with closed-ended, multiple-choice items regarding the number of patients seen per week, rehabilitation policy awareness (NRP, NHI, UNCRPD); and included a 23-item; 5-point Likert scale for nature of feedback, service equity, NHI awareness, Public-Private Practice (PPP) awareness.

**Data analysis:** for the qualitative data, the audio-recorded interviews were transcribed verbatim, with the resulting transcripts then read in conjunction with the audio recording to verify any transcription errors. Data were analysed using inductive thematic analysis according to Braun and Clarke, 2006 which entails the identification and reporting of patterns or themes revealed by the collected data. A theme, in this context, is something that reflects patterned responses within the data. A bottom-up approach in which themes found in the data are compared with the theoretical themes of the research study was undertaken [16]. The survey's quantitative data were analysed using descriptive statistics in IBM SPSS Statistics software version 28.0. Some open-ended responses to survey questions were recoded into categorical groups. The survey's recruitment was intended to augment the qualitative results of the interviews rather than to establish a representative, generalisable sample. Missing data was consequently omitted from the denominators when computing the response percentage to a question. The quantitative and qualitative data were merged at the presentation level by identifying the themes that could benefit from the integration of both types of data. A joint display of the results/findings was created using tables.

**Ethics approval and consent to participate:** this study was approved by the University of KwaZulu-Natal Biomedical Research Ethics Committee (approval number: BREC/000001338/2020). The study participants also signed a consent form before participating in the study.

## Results

**Demographic characteristics of the study participants:** Table 2 shows that the age of participants ranged from 23 to 56 years (median=29 years, IQR=12.5 years). Of the 99 participants (both quantitative and qualitative), 73 (74%) were female and 26 (26%) males. Of the 99 participants, 77 (78%) spoke isiZulu as their home language, with the remaining 22 (22%) spread between English, Afrikaans, and other languages. Up to 87 (88%) participants had an Honours degree (4-year bachelor´s degree) as their highest qualification. Approximately 71 (71%) of the participants were from the public sector, and 2 (2%) of the participants worked in a hybrid setup.

**Table 2:** demographic characteristics of study participants (N=99)

Age		Statistic
Mean		32.1
**Median**		**29.0**
Std. Deviation		8.6
Minimum		23
Maximum		56
Interquartile range		12.5
**Gender**	**Frequency**	**Percent**
Female	73	74
Male	26	26
**Home language**		
IsiZulu	77	78
English	14	15
Afrikaans	5	5
Other	3	3
**Highest qualification**		
Three-year degree	5	5
Four-year degree/honors	87	88
Postgraduate Diploma	2	2
Master’s degree	5	5
**Health sector**		
Public	71	71
Private	26	26
Hybrid	2	2

**Medical rehabilitation services availability:** one of the ways through which perceptions on the availability of rehabilitation services were gathered was by determining the extent to which the respondents felt that their facilities were situated in an area where rehabilitation service was most needed. The median score on this factor was 3.0 (interquartile Range (IQR=1.0)) on the 4-point scale and is indicative of a largely positive sentiment regarding rehabilitation services being situated where they are needed. The majority, that is, 42 rehabilitation facilities (61%) were available at the tertiary level compared to the primary level (Table 3).

**Table 3:** availability of rehabilitation services in eThekwini Metropolis, King Cetshwayo District Municipality, and the Amajuba District Municipality

Theme	Survey (n =42)			FGDs and interviews (n=57)	Meta-inference
Availability of rehabilitation services	**Level of care**			"…in Khombe there is only a speech therapist who is a com serve, all the other rehab services are not available...the community of Khombe does not have access to rehab services," Manager, Policy/Intelligent Development.	Rehabilitation is only available at the tertiary hospital level, both public & private. Rural areas have no service available. Local-level rehabilitation depends on CRB and periodic clinic rehabilitation team visits.
		**Public**	**Private**		
	Primary level (32%)	12%	10%		
	Tertiary level (78%)	61%	17%		
	**Geographic distribution of service**				
	Rural (45%)	45%	0%		
	Urban (55%)	38%	17%		

FGDs: focus group discussions

**Accessibility, effectiveness, and efficiency of medical rehabilitation services:** Table 4 presents a joint display of both quantitative and qualitative findings on the accessibility, effectiveness, and efficiency of medical rehabilitation services in the three study settings. On accessibility, the participants tended to disagree with the notion that most of their clients lived not more than 14 kilometers from the place offering rehabilitation services (Median=2.0, IQR=1.8). The participants were also not convinced that transport routes from surrounding areas were safe and reliable for clients to reach the rehabilitation facilities. Altogether, the overall rating on accessibility was subdued (median=2.0, IQR=2.0). Concerning the effectiveness of medical rehabilitation, study participants were asked to indicate the extent to which they agreed or disagreed with the statement, *“Rehabilitation programmes at my setting almost always do what they are intended to”*. The median score of 3.0 (IQR=1.0) indicates that the respondents´ general sentiment was positive regarding the effectiveness of the rehabilitation programmes provided in their settings. About the efficiency of rehabilitation services, the study participants were asked to indicate the extent to which they agreed or disagreed with the statement *“The results attained by my setting´s programmes are proportionate in terms of effort expended, money spent, resources used, and time utilised”*. The average median score of 3.0 (IQR=1.0) indicates that the respondents´ general sentiment tended to be positive regarding the efficiency of the rehabilitation programmes provided in their settings.

**Table 4:** accessibility, effectiveness, and efficiency of rehabilitation services in eThekwini Metropolis, King Cetshwayo District Municipality, and the Amajuba District Municipality

Theme	Survey					FGDs and Interviews	Meta-inference
Accessibility	**Distance to service location**					"...there is lack of access to care I believe because sometimes you have someone who wants access, they want to go to hospital but those barriers we keep talking about, finances and all of those things”. Manager, Control/Monitoring and Evaluation. “Maybe they have a problem with transportation; they live far away they have to borrow a vehicle from the community all of those things until they reach the hospital". Practitioner	Rehabilitation is not accessible to public sector patients. Private rehabilitation clients have access as it is tied to financial ability. Public rehabilitation in KZN requires extra out-of-pocket costs for transport; hospitals are usually in urban areas, distant from townships and rural areas.
	36 (85.7%) of the respondents disagreed their clients do not live more than 14 km away (median=2.0, IQR=1.8)						
	**Transport services to the location**						
	32 (76%) of the respondents agreed that transport routes from surrounding areas are safe and reliable for clients to reach their setting (Median=2.5, IQR=1.0)						
	**Overall access:** the overall rating on accessibility was subdued (Median=2.0, IQR=2.0).						
		**Public**	**Private**	**Rural**	**Urban**		
	**Median**	2.0	2.5	2.0	2.0		
	**IQR**	1.6	1.0	1.6	2.0		
Effectiveness	**Do rehabilitation programmes in your setting almost always do what they are intended to do?**					" Another thing on top of that is also the department how they categorise us, they call us support. If we are support, who are we supporting? We are diagnosing, we are treating, we are reducing disabilities so is that not important?" Practitioner 3	Rehabilitation in public health has poor effectiveness and efficiency compared to the private sector. Public rehabilitation clients have poor treatment completion rates, they do not re-intergrade optimally in society, and recovery is associated with complications. Delayed treatment and long waiting periods leave patients despondent and prematurely halt treatment.
	The median score of 3.0 (IQR=1.0) indicates that the respondents’ general sentiment tended to be positive regarding the effectiveness of the rehabilitation programmes provided in their settings.						
		**Public**	**Private**	**Rural**	**Urban**		
	**Median**	3.0	3.0	3.0	3.0		
	**IQR**	1.5	1.0	2.0	1.0		
Efficiency	**Are the results attained by my setting’s programmes proportionate in terms of effort expended, money spent, resources used, and time utilised?**						
	The general sentiment of the respondents tended to be positive regarding the efficiency of the rehabilitation programmes provided in their settings (median score 3.0, IQR=1.0).						
		**Public**	**Private**	**Rural**	**Urban**		
	**Median**	3.0	3.0	3.0	3.0		
	**IQR**	1.0	0.0	1.5	1.0		

IQR: interquartile range

**Affordability and equity of medical rehabilitation services:** as part of evaluating the affordability of rehabilitation services, the participants were asked to indicate the extent to which they agreed or disagreed with the statement *“The level of care we provide can be afforded by most clients in the community where my setting is situated”*. The mean score of 2.9 (SD=0.7) indicates that the respondents´ general sentiment tended to be mixed but with a skew towards agreeing that the services were indeed affordable. About equity, the quantitative exploration of views regarding equity involved asking the study participants to indicate the extent to which they agreed or disagreed with the statement *“My setting´s services are available to all members of the community who most need it, regardless of their social class”*. The median score of 2.9 (IQR=0.5) indicates that the respondents´ general sentiment tended to be mixed but with a skew towards agreeing that the services were indeed provided without any social class biases (Table 5).

**Table 5:** affordability and equity of medical rehabilitation services in eThekwini Metropolis, King Cetshwayo District Municipality, and the Amajuba District Municipality

Theme	Survey (n=42)					FGDs and Interviews (n=57)	Meta-inference
Affordability	The mean score on affordability was 2.9 (SD=0.7) indicating that the respondents’ general sentiment tended to be mixed but with a skew towards agreeing that the services were indeed affordable.					“There’s such a huge discrepancy between public and private and if someone can come and say here are the similarities, I don’t see them. I believe the only similarities are the physical personnel and the resources in terms of the human resources....other than that there’s absolutely nothing. It’s not equal, not at all; if I can’t go to private then I have to go to public. This means that automatically I know I will receive deteriorating service because of the huge barriers and hindrances that are-" Practitioner "There are different categorisations, you have your H0 patients who don’t pay, H1, H2, and H3”. G1 G2: “H3 patients are usually on medical aid but it’s still the cost is still lower compared to the assistive devices in private, so it’s like nothing. Practitioner	Affordability of rehabilitation linked to financial ability. Private health is unaffordable for most rehabilitation clients due to high service costs. Public rehabilitation in KZN is almost free. However, public health clients experience high out-of-pocket costs associated with rehabilitation service access, such as transport costs.
		**Public**	**Private**	**Rural**	**Urban**		
	**Mean**	3.1	2.7	2.9	2.9		
	**SD**	0.6	0.7	0.8	0.7		
Equity	The median score of 2.9 (IQR=0.5) indicates that the respondents’ general sentiment tended to be mixed but with a skew towards agreeing that the services were indeed provided without any social class biases						
		**Public**	**Private**	**Rural**	**Urban**		
	**Median**	3.0	3.0	3.0	3.0		
	**IQR**	0.0	1.0	0.0	2.0		

IQR: interquartile range

**Quality of medical rehabilitation services:** the practitioners´ perceptions regarding the quality of rehabilitation provided were partly assessed through the rating of several statements (Table 6). Cognisant of the fact that this was akin to a self-assessment, the participants were largely positive in their rating of the quality of service provided, with an overall median score of 3.0 (IQR=0.4) out of 4.0.

**Table 6:** quality of rehabilitation services in eThekwini Metropolis, King Cetshwayo District municipality, and the Amajuba District Municipality

Theme	Survey (n=42)					FGDs and Interviews (n=57)	Meta-inference
Quality medical rehabilitation services	**Practitioners provide a quality service in my setting**					“...a person with a disability is a priority but the person who manages people with disabilities is not prioritised.” Manager, Implementation /Service Provision “Other times in the private sector you must know which medical aid will pay or not pay you. And so, you will limit the service you give the patient. You’re not going to do your best. You’ll just say, ‘let me sacrifice 20 minutes only’; you could’ve done an hour.” Practitioner. “We could do more if we were more staffed; then we could do better because I think you’ve got physiotherapist and OT can work together very nicely because they literally on top of each other; whereas speech therapy is right on the other side of the hospital, but so it’s not close in terms of department structure.” Practitioner.	High-quality rehabilitation services in KZN are only available in private health. Private rehabilitation in KZN has an MDT protocol, an admission and discharge plan, and a review of the patient's post-discharge progress. Public rehabilitation has poor, compromised quality due to staff shortages; low priority placed rehabilitation by DoH; inadequate quality control measures, and limited resources due to budgetary constraints for rehabilitation.
	36 (85.7%) of the 42 respondents agreed the practitioners provide quality services (Median=3.0, IQR=1.0)						
	**The service we deliver at my setting is of good quality**						
	34 (81%) of the 42 respondents agreed that they deliver good quality services in their setting (Median=3.0, IQR=0.3)						
	**Our rehabilitation setting’s practices are in line- with the most current quality assurance standards of its peers**.						
	32 (76%) of the 42 respondents agreed that their practices are in line with the most current quality assurance standards (Median=3.0, IQR=0.0)						
	**Personal feedback from patients is generally positive**						
	33 (79%) of the 42 respondents indicated that feedback from their patients is generally positive (Median=3.0, IQR=1.0)						
	**The quality of rehabilitation service provided by my setting is of good quality**						
	33 (79%) of the 42 respondents indicated that services provided by their setting are of a good quality (Median=3.0, IQR=1.0)						
	**Overall quality:** the respondents were largely positive in their rating of the quality of service provided, with an overall median score of 3.0 (IQR=0.4) out of 4.0						
		**Public**	**Private**	**Rural**	**Urban**		
	**Median**	3.0	3.2	2.8	3.2		
	**IQR**	2.7	2.8	2.4	2.9		

IQR: interquartile range, FGDs: focus group discussions

## Discussion

In KZN, rehabilitation services are ostensibly available at all levels of care. A minority of clients utilise private healthcare rehabilitation, whereas public rehabilitation services are used by over eighty percent of the population [17]. Primary healthcare (PHC) level rehabilitation should comprise comprehensive care and prioritise those most in need [3]. Although rehabilitation services are available at the community level, they largely depend on community health workers (CHW); as attested to by this exchange between the researcher and a district health manager. *“Manager: ´we start at the community level with our rehabilitation services, whereby we now have community healthcare workers (CHWs). ´Researcher: ´when you say CHWs, do you mean they are therapists?´ Manager: ´no, they are CHWs´*. Community healthcare workers are untrained in recognising patients at risk for complications [3]. Regarding the CHW function, there are several viewpoints on their job title and identity, supervision, compensation, career opportunities, and psychological and emotional concerns [18]. A formal growth pathway or formal training to match CHWs with the National Qualifications Framework are just two of the elements missing from the current CHW program, even though the National Community Health Worker Profile Framework was developed for the CHW program [18]. Community Health Workers lack knowledge and skill in managing disabilities, especially childhood disorders [19].

Public clinics receive minimal rehabilitation in the form of outreach visits; as a district rehabilitation manager in KZN states, *“They (therapists) are in the hospital, and then they have to do outreach in CHCs that are linked to that hospital. In some places, it does happen, depending on whether they (therapists) have resources.”* Essentially, rehabilitation services in KZN are available at the tertiary level of healthcare, in hospitals that are mostly in urban areas, remote from rural areas [17]. Although South Africa´s public health system purportedly functions under PHC, rehabilitation services are available exclusively at District hospitals [17]. Equitable access to rehabilitation at the PHC level depends on a health system that provides services that are appropriate, affordable, and accountable [20,21]. As a developing, lower-middle-income country, access to equitable quality healthcare in South Africa is often linked to socioeconomic status and context [12,14,22,23]. As the burden of disability and non-communicable diseases becomes more rural, rehabilitation services are most needed in underdeveloped areas. The province of KZN is largely rural, with most patients having to travel long distances to access rehabilitation services in hospitals situated in urban areas [12,14,22,23]. A practitioner in the study pointed out that *“I think the problem is the location of the CHC…accessibility; because they are not serving a purpose you can´t build a CHC for people of Madadeni, but you built near Mathukuza…[it] is very far, obviously. The only transport that they can use is the one that takes them from Madadeni to town, so that´s the main problem. They have a beautiful setup, but they're not working for the patients.”*

Quality rehabilitation is determined by a comprehensive, patient-centered program [20]. For the South African health department, rehabilitation services are not a priority in practice. Rehabilitation services are not always offered within a bio-psychosocial model/framework but are persistently biased towards biomedicine, where doctors have authority over eligible patients [12,24]. A practitioner from the Amajuba district stated the following: *“… but I think the challenge we are having as rehab is to report to the medical managers who are doctors by qualification. So, the order of priority is not the same…when it comes to posts…; you´ll find that every department must say their needs, but then they talk to the doctors first.”* This lack of priority to rehabilitation is central to the poor quality of public rehabilitation services in KZN.

Lack of rehabilitation prioritisation discredits service quality by restricting and limiting human resources needed for optimum service provision in KZN. South Africa´s public health system is over-burdened with many patients and fewer rehabilitation practitioners. A rehabilitation manager spoke at length regarding this issue: *At the moment, to be honest, the government is experiencing financial constraints, so there is a bit of a strategy to minimise the expenditure with regard to the composition of employees. So, the department is capping; so currently what is happening, when a post is vacated it´s not automatically filled. Sometimes it happens but for rehab at the moment they are not on the exempted clinical lists. These exempted clinical lists, are doctors, pharmacists, radiographers, and nurses who have been put separately to say if that post is vacated you immediately fill it. However, rehabilitation therapists are on the list called non-exempted*. Minimal rehabilitation personnel led to patients receiving services late or given later and fewer appointments to elevate the backlog in KZN [12,14,24]. Patients in rural KZN grow despondent with long waiting times for rehabilitation sessions; they default on treatment and are thus lost in the system. Community rehabilitation workers (CRWs) is a clutch used by public health to cover chronic staff shortages due to a lack of prioritisation; however, CRWs cannot substitute therapists [3]. Patients resort to CRWs because it is the only service available to them at the local level; however, they are referred prematurely, and CRWs are untrained to identify risks that may lead to complications [3,12,14,17,24,25].

As a result of South Africa´s government placing minimal priority on rehabilitation, quality is interrupted by poor infrastructure and multidisciplinary practice. This study has shown that public hospitals are not always organised to assist easy multidisciplinary teams (MDT) practice; as one discipline is located on an opposite end from another [14]. In KZN there is little to no MDT practice; a practitioner from the King Cetshwayo District stated *“there are professionals, but they are not working together as a team. They are working in silos.”* The lack of rehabilitation personnel in KZN means that MDT practice is different from hospital to hospital, minimal, and has incomplete complements of rehabilitation disciplines. A practitioner in King Cetshwayo district attributed this to the Department of health´s bias towards the medical disciplines [12,14,21,23,26]. *“However, based on my experience in public and in private as well, they are working in exclusion. I believe their exclusion is not necessarily a lack of knowledge, but it has to do with- I am sorry to use this word- but a lot of ignorance and arrogance as well. They know the value of the rest of the multidisciplinary team, but you refuse to refer appropriately, and that gives huge negative outcomes to the patient. If you see a patient with stroke like you said it doesn´t matter if they have difficulty breathing it´s a stroke patient, we were also trained on how to manage that…”*

Similar to other rural settings, quality rehabilitation is available to those who can afford it [11,22]. Public sector rehabilitation in KZN is under-resourced, under-staffed, and has poor infrastructure; however, it must provide service to 80% of the province´s population. Private sector rehabilitation in KZN is costly and clients form an extreme minority of total users in the province. Affordable, resourced, and well-staffed rehabilitation with a comprehensive team of disciplines is available to those who can pay for it [11,22]. Lack of infrastructure, chronic personnel shortages, and poor rehabilitation quality lead to an ineffective and inefficient system overall in KZN. Practitioners in the current study were nearly unanimous in their negative assessment of the efficiency of KZN´s rehabilitation service system. The main issue is the lack of rehabilitation prioritisation by the South African government. This lack of priority placed on rehabilitation has resulted in little research in South Africa on rehabilitation governance and regulation [17]. One area that lacks regulation is monitoring and evaluation; systems are still paper-driven, and there is no meaningful rehabilitation data collection, resulting in practices not based on evidence [23]. As evident by our study, over 25% of all rehabilitation service providers stated that they do not keep records. Referral pathways are a further area of notable concern with rehabilitation in KZN. No set policy ensures a clear referral path and continuity of care at all stages of care [3].

Rehabilitation as PHC needs to be operationalised in KZN. Innovation is required to provide rehabilitation at the District Health System level to decrease and localise the number of final decision-makers regarding budget and policy [24]. Latest research (mostly in the Western Cape) indicates the promising initiation of Intermediate Care (IC) facilities [3,17]. Intermediate care facilities are step-down centers where the quality of life, and functional status is delivered by an MDT team at a level lower than that of acute care [3,25]. No such facility exists in the public sector in KZN. Symptoms or diagnostics related to rehabilitation (arthritis, spinal cord injury, head injury, neuromuscular disorders, stroke, fractures, and amputations) need to be classed in related groups of beta-scoring [3]. This district-based data will ease care planning for context-based, individualised programs [3,20].

## Conclusion

This research offers a comprehensive snapshot of the state of rehabilitation services in KwaZulu-Natal, which can serve as a foundation for policy development and implementation. Addressing the disparities in accessibility, particularly in rural areas, and building upon the positive aspects of affordability, equity, and service quality can further strengthen rehabilitation services, benefiting the diverse population of the province.

### What is known about this topic

- *The National Rehabilitation Policy and the UN Convention on the rights of persons with disabilities are playing a crucial role in shaping the landscape of rehabilitation services in South Africa;*
- *The importance of interdisciplinary collaboration in delivering comprehensive rehabilitation services has been highlighted by previous studies*.

### What this study adds

- *This study provides insights from a diverse range of stakeholders, including rehabilitation practitioners, managers, and social development representatives, offering a comprehensive understanding of the challenges and opportunities in rehabilitation services provision in South Africa;*
- *This study´s findings highlight areas for improvement and inform targeted interventions to enhance the quality and accessibility of rehabilitation services in South Africa;*
- *Our study adds evidence to the growing literature to support interventions for rehabilitation in South Africa and beyond*.
